# Embedding Systems Engineering Leadership in Learning Health Systems: The Case for a Chief Systems Engineer in Every Hospital

**DOI:** 10.1002/lrh2.70054

**Published:** 2025-12-05

**Authors:** Md Doulotuzzaman Xames

**Affiliations:** ^1^ Grado Department of Industrial and Systems Engineering Virginia Tech Blacksburg Virginia USA

**Keywords:** chief systems engineer, healthcare leadership, learning health systems, organizational learning, systems engineering

## Abstract

As healthcare systems transition toward Learning Health Systems (LHS), the need for executive‐level leadership in systems thinking becomes urgent. This commentary advocates for the institutionalization of a Chief Systems Engineer (CSE) role in hospitals to embed systems engineering (SE) principles within leadership structures, navigate complexity, integrate technology with clinical workflows, and drive continuous organizational learning. Drawing on lessons from high‐reliability industries and existing healthcare initiatives, this commentary argues that establishing dedicated SE leadership is essential for achieving both horizontal and vertical integration across clinical, operational, and strategic domains. It outlines the responsibilities of the CSE, aligns them with LHS goals, and offers recommendations for sustained and scalable implementation across healthcare organizations.

## 1 Introduction

Although advancements in clinical practice, diagnostics, and digital technologies have been transformative, the underlying delivery systems remain fragmented, inefficient, and vulnerable to cascading failures. These challenges are not merely operational; they are systemic. Addressing them requires the integration of systems engineering (SE), a discipline focused on the design, coordination, and optimization of complex systems across their life cycles. By aligning people, processes, technologies, and data, SE provides the foundational methodology needed to build adaptive, resilient, and learning‐oriented health systems.

Healthcare organizations have increasingly demonstrated the promise of SE through collaborative projects between engineers, clinicians, and administrators. Yet, these efforts often remain localized—project‐based initiatives that improve parts of the system without reshaping the system as a whole. This distinction is central: SE can be practiced as an activity without a dedicated leadership role, but its long‐term institutional impact depends on whether systems thinking is embedded within executive decision‐making structures.

The global health community increasingly embraces the paradigm of Learning Health Systems (LHSs) [1, 2]. By definition, LHSs rely on feedback‐driven adaptation, continuous improvement, and system‐level learning, challenges that are inherently suited to SE methods. To realize this potential, however, SE must evolve from a set of tools applied intermittently to an organizational function governed strategically. This commentary, therefore, argues not that SE cannot occur without formal leadership, but that its transformative capacity is limited in the absence of sustained systems oversight at the executive level.

Accordingly, this paper advances a bold yet pragmatic proposition: every hospital should embed a Chief Systems Engineer (CSE) within its executive leadership team. The CSE would not replace existing SE or quality improvement (QI) initiatives but rather provide enduring stewardship over their alignment, integration, and learning. By institutionalizing SE principles at the leadership level, the CSE can bridge technical infrastructure, clinical workflows, organizational processes, and learning mechanisms in pursuit of the adaptive, equitable, and resilient vision of an LHS.

## 2 The Imperative for Systems Engineering in Learning Health Systems

LHSs are characterized by their ability to seamlessly integrate data and evidence into care delivery, adapt practices based on real‐time outcomes, and institutionalize feedback loops for continual refinement and improvement [3]. These goals, while noble and necessary, are complex in practice. Implementing an LHS demands not only digital infrastructure and data science capabilities but also the intentional design of workflows, stakeholder engagement strategies, information pathways, and governance structures: tasks that are fundamentally within the domain of SE [4].

As defined earlier, SE provides a structured discipline for coordinating people, processes, technologies, and data across the full lifecycle of a complex system [5, 6]. Its methods emphasize eliciting and prioritizing stakeholder needs, translating them into measurable requirements, and designing integrated solutions that maintain coherence and traceability throughout implementation and operation. This requirements‐driven orientation differentiates SE from incremental improvement efforts and aligns directly with the long‐term goals of LHSs.

Healthcare systems have made important strides in QI through methodologies such as Lean and Six Sigma [7, 8]. These approaches, alongside frameworks that emphasize patient co‐production and continuous learning, have significantly shaped local practices [9, 10, 11]. However, QI initiatives often remain bounded by departmental or process‐level scopes and thus fall short of achieving the system‐wide integration required for sustainable learning. SE complements QI by addressing design and coordination at the enterprise level—ensuring that improvements across units align with shared objectives, dependencies, and constraints. Rather than competing with QI, SE enables its insights to propagate and compound, providing the architecture through which isolated improvements coalesce into organizational transformation.

Despite its demonstrated promise, SE in healthcare still functions largely as a project toolkit rather than a strategic framework [12]. Institutionalizing a CSE role would shift SE from a set of ad hoc methods to a continuous organizational capability. Additionally, embedding SE leadership in healthcare provides the governance structure needed to operationalize the vision of an LHS.

## 3 Lessons From High‐Reliability Safety‐Critical Domains

The utility of SE leadership has long been recognized in sectors where failure has catastrophic consequences. In aerospace, nuclear power, and defense, systems engineers occupy critical leadership roles tasked with ensuring integration across complex subsystems, maintaining safety, and optimizing performance under uncertainty. NASA institutionalized SE leadership following the Apollo 1 disaster, leading to profound improvements in risk management and organizational coordination [13, 14]. Similarly, the U.S. Department of Defense mandates SE processes throughout the acquisition lifecycle of major defense systems [15].

Healthcare is a complex adaptive system [16] and is arguably no less safety‐critical [17]. Yet, unlike those high‐reliability industries, healthcare rarely embeds SE leadership at the executive level. QI initiatives have achieved significant process‐level gains, and some have embraced elements of systems thinking—what Williams and Best [18] describe as efforts that “range from point‐of‐care projects to whole‐system change,” with the latter remaining elusive. Their analysis underscores that leadership, organizational structure, and culture are essential preconditions for moving from isolated improvements toward an integrated systems approach. This evidence reinforces the argument for SE leadership: systems engineers provide the very integrative architecture and lifecycle perspective required to achieve the system‐level coherence that QI practitioners aspire to but often cannot sustain at scale.

The absence of a dedicated systems‐level integrator within hospital leadership contributes to what may be termed “organizational fragility”—a condition in which interdependent subsystems, such as clinical, technical, and administrative domains, lack the coordination mechanisms necessary to absorb shocks, adapt to change, or sustain improvement. In practice, such fragility manifests as redundant initiatives, conflicting priorities, and brittle processes that falter under stress. Introducing a CSE can mitigate these risks by transforming fragmentation into resilience—ensuring that improvement efforts are integrated rather than siloed, sustained rather than episodic, and systemic rather than isolated.

Although no single role can resolve all challenges, the CSE can orchestrate cross‐disciplinary learning, bridging technical expertise, operational management, and clinical insight. Through this role, healthcare systems can institutionalize the same disciplined integration that has underpinned safety and reliability in other complex domains, enabling a transition from reactive adaptation toward proactive system design and continual learning.

## 4 The Unique Role and Value Proposition of the Chief Systems Engineer

The CSE represents a new category of executive leadership in healthcare—one focused on the design, coordination, and continuous evolution of the hospital as a complex sociotechnical system. Whereas most existing executives oversee particular operational or functional domains, the CSE's mandate is explicitly integrative: to align clinical, operational, technological, and organizational subsystems toward shared strategic objectives.

This role is distinguished not by managing daily operations but by engineering the interfaces among them. The CSE can act as the institutional architect of coherence—ensuring that digital innovations, workflow redesigns, and policy changes reinforce rather than counteract one another. By combining SE rigor with healthcare insight, the CSE can transform isolated initiatives into components of a unified learning architecture that supports the goals of an LHS.

Specific responsibilities of the CSE include:

- Designing and continuously refining adaptive workflows that enable safe, evidence‐based, and data‐driven care.
- Integrating digital and analytical tools into clinical practice in ways that are usable, transparent, and ethically sound.
- Institutionalizing feedback loops that convert frontline experience and data into organizational learning.
- Coordinating across departments to minimize redundancy, align resources, and build resilience against system shocks.
- Guiding the evaluation and scaling of innovations so that lessons learned in one area propagate system‐wide.

In fulfilling these responsibilities, the CSE can bridge leadership silos without displacing them—working collaboratively with operations, information, and clinical executives to ensure that improvement efforts are structurally compatible and strategically aligned. The value of the role lies in its ability to transform strategic intent into engineered capability: to move the organization from reactive problem‐solving toward proactive system design.

## 5 Emerging Models and Evidence of Impact

Although the CSE role is not yet commonplace, promising prototypes of SE leadership are emerging. The U.S. Veterans Health Administration (VHA) has demonstrated measurable benefits from integrating SE principles into healthcare redesign—improvements in patient flow, care coordination, and staff satisfaction have been documented in pilot programs [19]. These initiatives represent early forms of horizontal diffusion: the spread of SE methods across projects, clinics, or facilities through collaboration and knowledge exchange. Although effective in specific contexts, such diffusion remains limited by the absence of an institutional mechanism to integrate learning vertically—linking project‐level insights to organizational governance and strategic decision‐making.

Collaborations between academic SE programs and healthcare institutions have also yielded innovative solutions to complex problems, including emergency‐response planning and telehealth optimization during the COVID‐19 crisis [20, 21]. However, these efforts typically arise from time‐limited grants or individual champions. Without formal leadership authority, lessons from such collaborations often fail to influence resource allocation, policy, or long‐term design decisions.

Institutionalizing a CSE can directly address this limitation by enabling vertical diffusion of SE across organizational layers. In this configuration, SE knowledge no longer flows solely laterally among technical teams but also upward into executive governance and downward into operational practice. The CSE can ensure that insights from frontline improvement inform strategic priorities, and that organizational strategy, in turn, cascades coherently through system design and implementation.

## 6 Alignment With the Core Principles of Learning Health Systems

The integration of a CSE into hospital leadership is not merely compatible with the LHS model—it is foundational to realizing it. LHSs depend on continuous data capture, feedback, and adaptation, yet these mechanisms falter without deliberate system design and sustained coordination. SE provides the architectural discipline that enables these feedback loops to function at scale: it ensures that data flows are interoperable, that insights are translated into implementable design changes, and that those changes are validated and refined through ongoing measurement [22].

QI programs typically emphasize iterative cycles of change within discrete units or processes, focusing on “what works here.” SE extends this logic by addressing “how it all fits together.” Rather than replacing QI, SE provides the meta‐structure through which local improvements can accumulate into system‐wide learning. This distinction positions SE—and, by extension, the CSE role—as the integrative mechanism that transforms multiple QI efforts into a coherent, self‐learning enterprise.

Through this lens, the CSE can serve as the institutional conduit that aligns the operational, informational, and strategic dimensions of the LHS. By overseeing the design of interoperable infrastructures and coordinating feedback governance, the CSE can ensure that lessons from clinical practice are encoded into system redesign and policy refinement. The role thus can transform LHS principles from conceptual aspirations into engineered processes—bridging data and practice, learning and governance, technology and human factors.

Moreover, as LHSs increasingly depend on digital infrastructure and algorithmic tools, they raise pressing concerns about transparency, bias, and accountability. Systems engineers, trained in systems thinking, human‐centered design, process optimization, and risk analysis, are uniquely positioned to ensure that such technologies are implemented ethically and equitably. In this capacity, the CSE functions as both a technical architect and an ethical steward—bridging innovation and implementation to sustain the trust and adaptability upon which LHSs rely.

## 7 Challenges and Recommendations for Implementation

Institutionalizing the CSE role will require overcoming several barriers. First, there is a lack of awareness among healthcare leaders regarding the full scope and strategic value of SE. SE is often conflated with IT management or process optimization, obscuring its broader organizational and lifecycle perspective. Second, uncertainty persists around optimal reporting structures, role definitions, and interfaces with existing leadership functions—issues that must be clarified to prevent redundancy and to secure executive buy‐in. Third, a shortage of professionals fluent in both SE principles and healthcare operations constrains immediate adoption.

Beyond technical competence, implementation will hinge on organizational culture and incentives. Hospital leaders may resist adding new leadership positions unless the role's contribution to efficiency, resilience, and staff well‐being is explicitly demonstrated. One pragmatic strategy is to frame the CSE not as additional management overhead but as a reallocation of existing capacity—repurposing current systems integration, quality, or analytics functions under unified SE leadership. By emphasizing measurable benefits—reduced duplication, improved resource alignment, and enhanced adaptability—the CSE position can be justified within existing governance and budget structures.

Payers and regulators can further accelerate adoption by linking performance metrics to organizational learning and resilience. Incentive programs that reward hospitals for cross‐departmental coordination, digital interoperability, or workforce sustainability could explicitly recognize SE leadership as a qualifying mechanism. Similarly, accreditation bodies might incorporate SE‐related competencies within leadership standards, reinforcing the notion that system integration is essential to quality and safety rather than ancillary to it.

To address these challenges, the following actions are recommended:

1. *Education and advocacy*: Professional and academic societies across SE and healthcare should collaborate to raise awareness of SE's strategic role and integrate core SE concepts into clinical management, public health, and health administration curricula.
2. *Workforce development*: New pathways, such as joint degrees, fellowships, and continuing‐education programs, should cultivate hybrid leaders skilled in both SE and healthcare delivery.
3. *Policy and incentives*: Regulatory and accreditation agencies can promote SE leadership by embedding systems integration requirements within institutional performance frameworks.
4. *Pilot programs and evaluation*: Hospitals should pilot the CSE role within defined subsystems, evaluate its impact on quality, resilience, and staff well‐being, and disseminate lessons learned to guide scalable adoption.

## 8 Conclusion

The evolution of LHSs depends not only on technology and data but on the deliberate engineering of healthcare as a coherent, adaptive enterprise. SE offers the methodological foundation for this transformation, yet its potential remains limited without leadership dedicated to sustaining integration across people, processes, and technologies. Embedding a CSE within hospital leadership translates SE from a project‐based activity into an organizational capability—linking operational learning with strategic governance and ensuring that adaptation becomes continuous rather than crisis‐driven. Establishing this role represents a practical step toward building health systems that are resilient, equitable, and capable of continuous learning.

## Conflicts of Interest

The author declares no conflicts of interest.

## Data Availability

Data sharing not applicable to this article as no datasets were generated or analysed during the current study.
